# Molecular basis of resistance to leaf spot disease in oil palm

**DOI:** 10.3389/fpls.2024.1458346

**Published:** 2024-12-09

**Authors:** Cahyo S. Wibowo, Ricki Susilo, Reza Ernawan, Ardha Apriyanto, Mohammed O. Alshaharni, Graham R. Smith, Angharad M. R. Gatehouse, Martin G. Edwards

**Affiliations:** ^1^ School of Natural and Environmental Sciences, Newcastle University, Newcastle upon Tyne, United Kingdom; ^2^ Research and Development, PT Astra Agro Lestari Tbk (Astra Agro), Jakarta, Indonesia; ^3^ Biology Department, College of Science, King Khalid University, Abha, Saudi Arabia; ^4^ Bioinformatics Support Unit, Faculty of Medical Sciences, Framlington Place, Newcastle University, Newcastle Upon Tyne, United Kingdom

**Keywords:** disease resistance, leaf spot, oil palm, nursery, RNA-Seq, *Curvularia oryzae*

## Abstract

**Introduction:**

Leaf spot disease caused by the fungal pathogen *Curvularia oryzae* is one of the most common diseases found in oil palm (*Elaeis guineensis*) nurseries in South East Asia, and is most prevalent at the seedling stage. Severe infections result in localized necrotic regions of leaves that rapidly spread within nurseries leading to poor quality seedlings and high economic losses.

**Methods:**

To understand the molecular mechanisms of this plant-pathogen interaction, RNA-Seq was used to elucidate the transcriptomes of three oil palm genotypes with contrasting pathogen responses (G10 and G12, resistant and G14, susceptible) following infection with *C. oryzae* spores. Transcriptomes were obtained from Illumina NovaSeq 6000 sequencing of mRNA at four different time points (day 0, before treatment; day 1, 7, and 21 post treatment).

**Results and discussion:**

Analysis of differentially expressed gene (DEG) profiles in these three genotypes provided an overview of the genes involved in the plant defence. Genes involved in disease resistance, phytohormone biosynthesis, gene regulation (transcription factors), and those encoding proteins associated with cell wall hardening were identified and likely contribute to the resistance of oil palm to *C. oryzae*. Such genes represent good candidates for targets to enhance oil palm productivity and resilience through molecular breeding approaches.

## 1 Introduction

*Elaeis guineensis*, oil palm, is a major commodity in tropical countries such as Indonesia and Malaysia. High quality edible vegetable oil is extracted from the mesocarp of the fruit. The oil is primarily used in food preparation and manufacturing but is also found in beauty products and biofuels; it accounts for up to 40% of traded vegetable oils. Oil palms are grown on plantations and trees remain productive for more than 25 years. The long-lived perennial crops use a much smaller land footprint compared to annual oilseed crops. However, as seen with all crops, oil palm productivity is threatened by both biotic and abiotic stress factors which hamper growth, development and impact yields. Pathogen-induced biotic stress, such as leaf spot disease, may cause significant palm loss especially when the infection occurs in nurseries due to incorrect disease prevention and management, as well as the use of susceptible genotypes. Leaf spot disease is a common phenotype caused by several fungal species mainly from the genera *Cercospora*, *Colletotrichum*, *Bipolaris*, and *Curvularia* (Kittimorakul et al., 2019). The pathogen infects oil palm seedlings, immature and mature palms and presents with typical symptoms of yellow necrotic spots on the leaves. As the disease progresses these lesions expand and turn brown as the leaf tissue dies. Outbreaks of leaf spot are common during the rainy season due to favourable environmental conditions for conidial spread and germination (Sunpapao et al., 2014; Kittimorakul et al., 2019). This emerging disease is commonly identified in small farmers’ nurseries, but also in the larger nurseries owned by large national and international companies. Unless treated correctly, the outcomes are poor quality seedlings leading to high economic losses.

Currently several control measures for the disease are required to overcome this crop loss (Sunpapao et al., 2018). Unfortunately, such measures result in significant additional costs for small farmers who would benefit more from growing disease-resistant genotypes. Preliminary screening of the Astra Agro Lestari (AAL) genotype collection has identified crosses with contrasting responses to *C. oryzae* infection and disease progression that would likely provide such resistant genotypes. Thus nursery screening has shown great potential to identify new resistant genotypes. Recently Kittimorakul et al. (2019) proposed that a single timepoint of 20-days after exposure to the pathogen could be used to identify tolerant genotypes within a population. However, observing the oil palm response to leaf spot disease over an extended period of infection would be useful to validate the temporal, and repeated, infection response and would provide a more authentic exposure timeframe as commercial seedlings are normally maintained in the nursery for at least 10-12 months. The area under the disease-progress curve (AUDPC) could be used as an additional criterion to distinguish genotypes demonstrating tolerance to disease stress throughout a period of continuous exposure to the pathogen (Jeger and Viljanen-Rollinson, 2001; Simko and Piepho, 2012; Schandry, 2017; McLean et al., 2022; Terao et al., 2022). AUDPC is frequently used in plant-disease studies based on the calculation of disease severity observed at several time points, the output being a single value for a straightforward comparative analysis. A further method for analysing plant response post disease stress for a longer period is Kaplan-Meier survival estimates (Soler et al., 2015). This statistical estimator can be used to evaluate the development of severe symptoms after treatment where a sharp decline in survivorship indicates the susceptibility of genotypes in breeding for disease resistance.

Apart from basic phenotypic observations, only a limited number of studies on plant-pathogen interactions in oil palm, and in particular leaf spot disease, have been carried out to date. Therefore, it is important to study the regulation of genes central to this plant-pathogen interaction. Utilization of RNA sequencing (RNA-seq) technology provides new possibilities to explore and describe contrasting responses of oil palm genotypes to leaf spot disease, thus elucidating the underlying mechanisms of host-plant resistance. Previous research also shows that this technology is important in decoding response mechanisms to leaf spot disease in maize (He et al., 2021) and cucumber (Sa et al., 2020).

The present study investigates the response of three different genotypes (G10 and G12, resistant; G14, susceptible), to leaf spot disease in nursery screening trials. The two resistant genotypes were selected based on previous studies (Wibowo et al., 2023), whilst G10 and G12 represent SP540 1 and SP540 2, respectively. Interestingly, the resistant genotypes have different genetic backgrounds, but exhibit similar levels of resistance to this disease.

In addition, the present study examines both phenotypic data from nursery screening and the expression patterns of DEGs between susceptible and resistant genotypes, shedding new light on both the understanding of the genetic basis of resistance/tolerance, and mechanisms of plant defence to leaf spot disease. This knowledge will help inform the breeding of disease resistant varieties for use by growers.

## 2 Materials and methods

### 2.1 Plant materials and experimental design

Three different oil palm (*E. guineensis*) genotypes derived from Deli x SP540, and Deli x (LaMe x SP540) origins were used for this study (see **Supplementary Table S1**). The experiment, carried out in triplicate, was organised in a split-plot design in environmentally controlled growth cabinets (Sanyo MLR-350, Japan). Temperature was set at 29°C day and 27°C night, with 9 hours photoperiod. One cabinet was used for inoculum treatment and the other one as control (non-treated) as detailed in **Supplementary Figure S1**. Oil palm leaves were inoculated with an aqueous suspension of *C. oryzae* spores (10^5^ spores/ml in sterilized water). Viability of spores delivered in this manner was > 70%. Spores were applied to test leaves using a fine homogeneous spray, inoculum was applied *ad libitum* until run-off was observed.

### 2.2 RNA sequencing

Total RNA from leaf samples was taken at day 0 (before treatment), 1, 7 and 21 post inoculum treatment using Norgen Plant/Fungi Total RNA Purification Kit (Norgen Biotek^®^) following the manufacture’s protocol. Three genotypes were selected for transcriptomics study (resistant genotypes G10, G12 and susceptible genotype G14) with four different time points and three individual samples (biological replicates) for each genotype. Total RNA was quantified using a NanoDrop spectrophotometer (Thermo Fisher Scientific, USA) and a Qubit RNA High Sensitivity Assay on a Qubit 2.0 Fluorometer (Life Technologies, USA). Total RNA quality was determined using an RNA Screen Tape on an Agilent Tape Station 4150 (Agilent Technologies, USA). The RNA samples were sequenced using the Illumina NovaSeq 6000 sequencing platform (Novogene UK). To guarantee the reliability of the data, quality control (QC) was performed at all stages, from RNA extraction to post-sequencing analysis. The sequenced reads were quality-checked using fastp software (Chen et al., 2018). The raw reads were trimmed using the Trimmomatic software (Bolger et al., 2014).

### 2.3 Data analysis

After quality assessment, transcriptomic analysis was conducted using a Rocket HPC with oil palm transcriptome and gene annotation from NCBI as reference. A quantification of RNA-seq data was performed using Salmon version 1.10.2 (Patro et al., 2017), and DESeq2 packages (Love et al., 2014) were then used for subsequent analysis in R version 4.3.1 (RCoreTeam, 2020) as well as NetworkAnalyst (Zhou et al., 2019). Genes were considered to be significantly differentially expressed if, in a given contrast, they had adjusted p.value ≤ 0.05 and |log2 (fold-change)| > 1. Relative gene expression of genes from the qPCR studies were assessed using a ΔΔCt method (Livak and Schmittgen, 2001). ΔCt values were calculated using the oil palm manganese superoxide dismutase (*MSD*) gene as the reference gene (Ooi et al., 2012; Kwan et al., 2016). The *MSD* gene was selected from the four candidate reference genes with RefFinder software (Xie et al., 2012), based on stability. RefFinder is a web-based comprehensive tool for evaluating and screening reference genes and it combines current computational programs (geNorm, Normfinder, BestKeeper, and the comparative ΔΔCt method) to compare and rank the tested candidate reference genes. A reference condition related to the respective experiment was chosen to calculate ΔΔCt values and subsequently elucidate a relative fold change. Student t-test was used to compare each treatment with the reference condition for significant difference between two treatments using ΔCt values.

### 2.4 Validation of RNA-seq using qRT-PCR

Primers (**Supplementary Table S2**) were designed to amplify sections of four housekeeping genes and eight target genes. Quantitative real-time PCR (qRT-PCR) was performed using a QIAGEN RotorGene 6000 (QIAGEN, Venlo, The Netherlands) in 20 µL reactions, using Bioline 2X SensiFAST SYBR^®^ No-ROX Kit Master Mix (Meridian Biosciences, Cincinatti, OH, USA), with primers at 400 nM final concentration. Three technical replicates were performed per sample. The PCR conditions were 2 min of hold at 95°C, followed by 40 cycles of 5 s denaturing at 95°C, 10 s annealing at 60°C and 15 s of extension at 72°C, with fluorescence acquisition at the end of every extension step. A melt curve was performed at the end of the run, increasing the temperature by 1°C from 72°C to 95°C, to evaluate the amplicon.

## 3 Results

### 3.1 RNA sequence analysis

RNA extracted was of high quality with an RNA integrity number (RIN) greater than 6.6 in all cases (mean 7.1, SD 0.2, **Supplementary Table S3**) and was acceptable for sequencing purposes in oil palm. Illumina NovaSeq 6000 sequencing generated 239.2 Gb of raw data. An initial data review indicated Q-scores ≥ Q30, thus indicating good quality sequencing had been obtained. The average GC content was recorded at 49.11%. Detailed statistics for the quality of sequencing data are shown in **Supplementary Table S4**.

**Figure 1:**
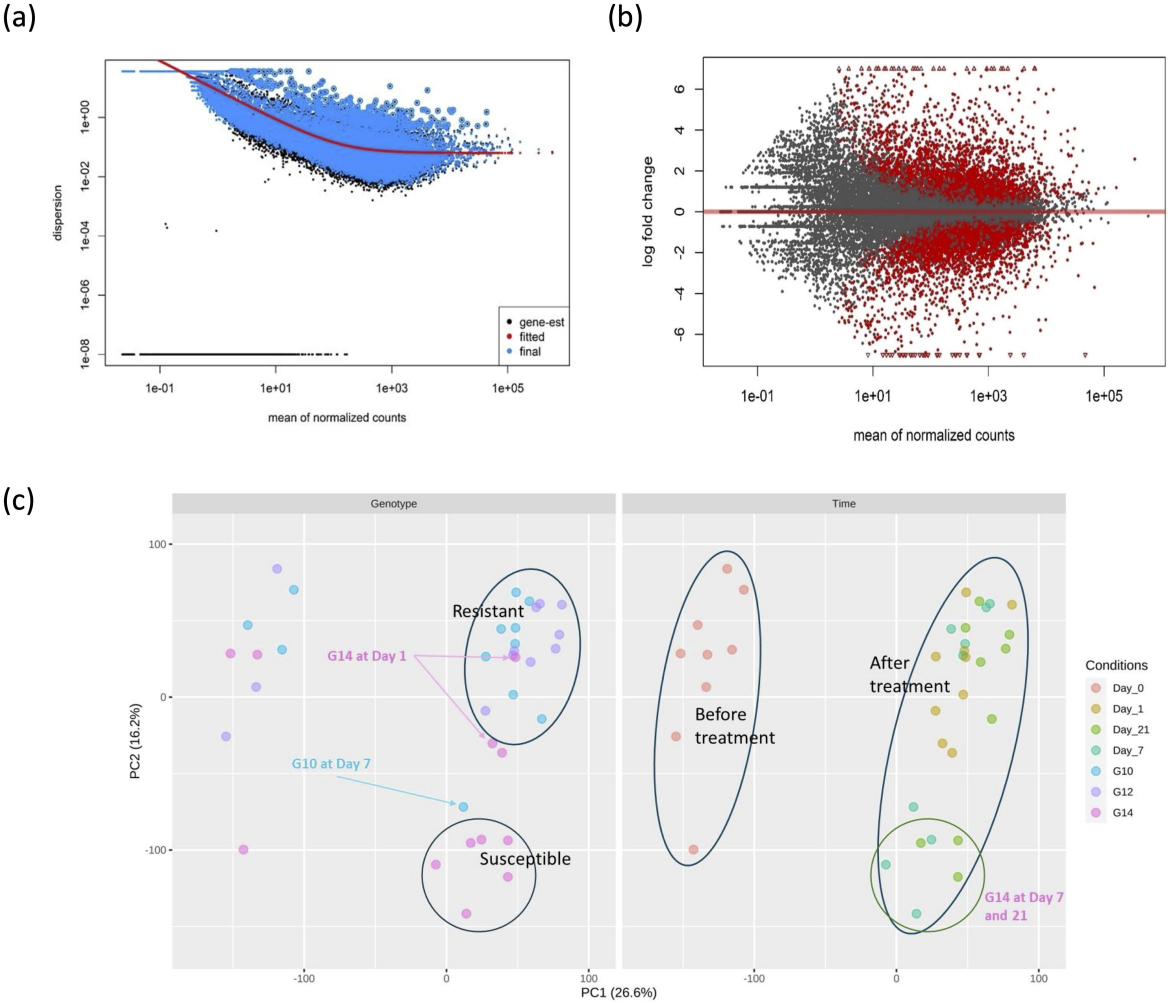
Differential gene expression and clustering analysis of susceptible and resistant oil palm genotypes in response to infection. **(A)** Dispersion plot produced by DESeq2 package. The dispersion variance decreased with increasing read count as expected in the typical RNA-seq experiment without or less deviation. **(B)** MA plot for alpha = 0.05 between G14 samples at day 1 after treatment and before treatment. The red dots above the red horizontal line denote up-regulated gene expression, the red dots below the red line denote down-regulated gene expression. Log2 fold change > 2.0 and adjusted p-value > 0.05 were used as threshold. **(C)** PCA plot comparing 36 samples in the study. The left and right figures are similar PCA analyses. The LHS figure represents a clustering based on genotype and the RHS figure represents a clustering based on time of treatment. All three genotypes clustered together before treatment. The susceptible genotype G14 clustered separately from the resistant genotypes at day 7 and day 21.

Prior to the identification of differentially expressed genes (DEGs), the sequence data was assessed with respect to an estimate of gene-wise dispersion and clustering of similar pools of samples. A diagnostic plot for dispersion estimates of this study is shown in **Figure 1A**, with most data scattered around the curve, as expected for RNA-seq studies where variance decreases with increasing read count. Results from the MA plot (**Figure 1B**) show that there is a significant difference in gene expression between G14 samples at day 1 post-treatment compared to pre-treatment (day 0), indicating that pathogen treatment significantly impacts gene expression. The PCA plot for gene expression (**Figure 1C**) indicated that the three genotypes (G10, G12 and G14) all clustered together prior to inoculation with the fungal pathogen; however, at day 7 and day 21 the susceptible genotype, G14, clustered separately from both resistant genotypes (G10 and G12) post treatment. One outlier, G10 at day 7, was identified out of 36 samples from three genotypes and four time points.

**Figure 2:**
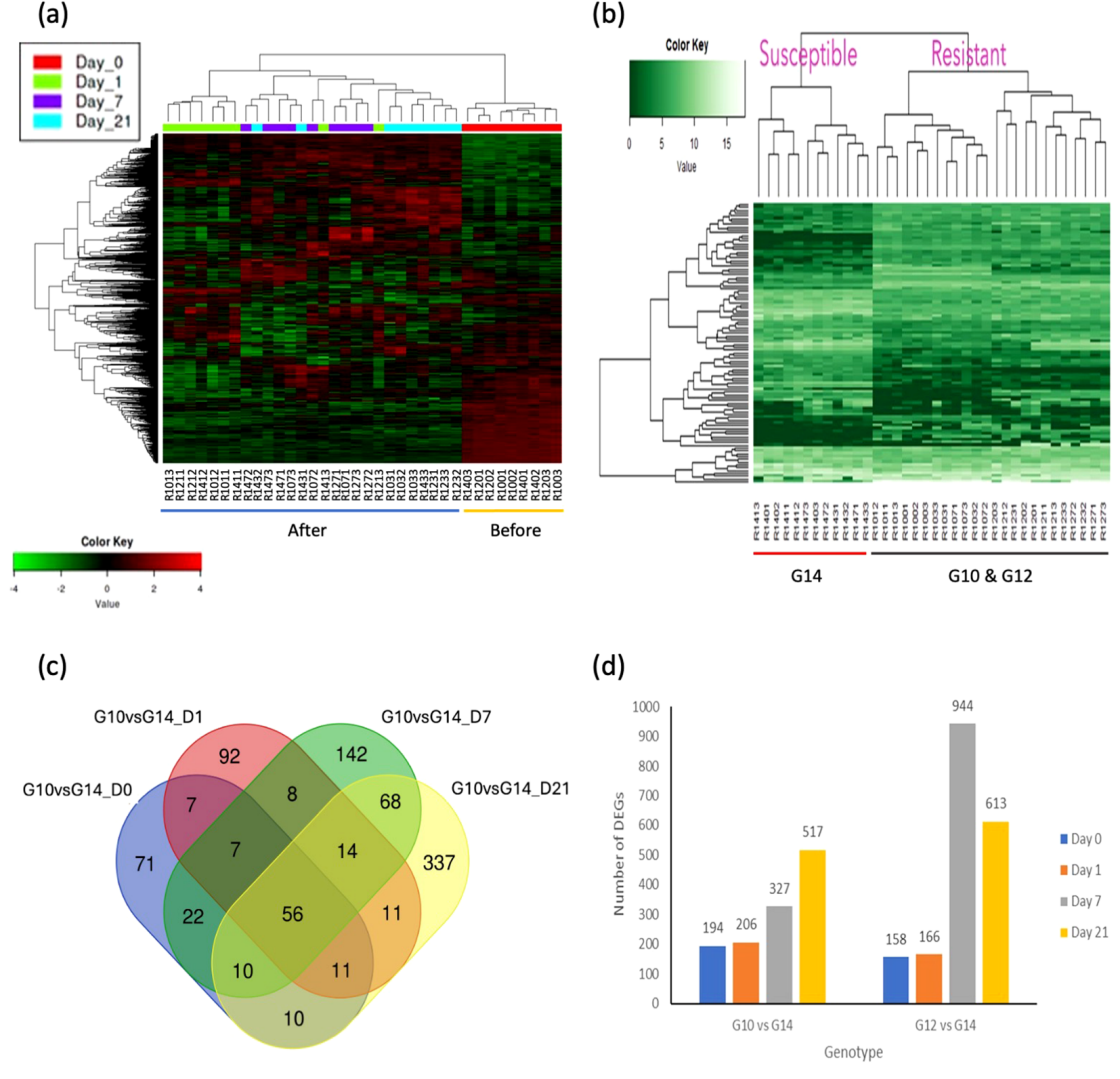
Heatmap analysis and differential gene expression (DEG) analysis between different oil palm genotypes and different times post treatment. **(A)** Heatmap analysis based on time of treatment. Two major groups were compared i.e. before treatment (Day 0) and after treatment (Day 1, 7, and 21). The samples clustered based on their corresponding expression profiles. Heatmap with symmetric diverging colour scale of log transformed count matrix values for 1,500 DEGs comparing samples before and after treatments. Red colour denotes up-regulated genes and green colour denotes down-regulated genes. Log2 fold change > 2.0 and adjusted p-value > 0.05 were used as the threshold. **(B)** Heatmap analysis based on oil palm genotypes. Two major groups were compared i.e. susceptible and resistant. The samples clustered based on their corresponding expression profiles. Single colour scale heatmap of log transformed count matrix values for 50 DEGs comparing resistant and susceptible genotypes. Lighter green colour represents higher gene expression levels and darker green indicates lower expression levels. Log2 fold change > 2.0 and adjusted p-value > 0.05 were used as the threshold. **(C)** Differentially expressed genes following *C. oryzae* infection between genotype G10 and G14 at different time points. At day 1 post treatment, the total number of differentially expressed genes (268 DE genes, green colour) was similar to day 0 (248 DE genes, red colour). Ninety-seven differentially expressed genes were common to both conditions. A higher number of DEGs were identified at 7 and 21 days after treatment (359 and 593 DEGs respectively) compared to day 0 (before treatment). Overlapping zones indicate a common response between genotypes, genes outside of the overlapping zones are unique to that genotype. Log2 fold change > 2.0 and adjusted p-value > 0.05 were used as the threshold. **(D)** Number of DEGs identified at each time point with DESeq2 package in NetworkAnalyst software for each genotype pairs comparison with susceptible genotype G14 used as a common control for both resistant genotypes. Number of DEGs for both comparisons increase at day 7 and day 21 post inoculation when compared to the susceptible genotype at a specific time point. Genotype G12 at day 7 post inoculation has the highest number of DEGs (944) when compared to genotype G14. Log2 fold change > 2.0 and adjusted p-value > 0.05 were used as threshold.

Differentially expressed genes and transcription factors were identified by DESeq2 package using the NCBI gene annotation reference by comparing gene expression between samples. Symmetric diverging colour scale heatmaps (**Figure 2A**) and single colour scale heatmaps (**Figure 2B**) were used to display higher and lower gene expression levels among 36 samples for 1,500 DEGs (between pre-and post-treatment comparisons) and the top 50 DEGs (between resistant and susceptible genotypes), respectively. The top DEGs were related to plant defence, cell-wall hardening, environmental stress and phytohormone synthesis.

RNA-seq analysis identified 172 DEGs between the three genotypes in the absence of infection (equivalent to time 0). Comparison between these three genotypes at all time points after pathogen infection relative to non-infected healthy plants (susceptible G14, Day 0) identified 1,802 DEGs. The top 50 DEGs revealed that higher gene expression levels occurred in both the resistant genotypes (G10, G12), compared to the susceptible genotype G14 (**Supplementary Table S5**). Interestingly, several of the highly differentially expressed genes encode proteins that are related to plant defence, such as RGA1, RGA2, RGA3, RGA4 (resistance gene analouges 1-4), PR-1 (pathogen-related protein 1) and CHS (chalcone synthase). A Venn diagram of DEGs between the resistant genotype G10 and the susceptible genotype G14 at different time points (**Figure 2C**) showed that the number of differentially expressed genes specific to the pre-treatment and day 1 post treatment were 71 and 92, respectively. In contrast, there was a higher number of DEGs identified at both 7 and 21 days post treatment, with 142 and 337 DEGs, respectively. Fifty-six DEGs were common to all conditions, irrespective of genotype (**Figure 2C**). A Venn diagram of DEGs between the resistant genotype G12 and the susceptible genotype G14 at different time points is presented in **Supplementary Figure S3**.

RNA-seq analyses were further refined whereby the two resistant genotypes were individually compared to the susceptible genotype over time, post inoculation. These analyses identified > 1,500 DEGs (**Figure 2D**). The complete list of DEGs between genotypes and each time point can be seen in **Supplementary Table S6**.

In addition to genes relating to plant defence, several genes were related to plant growth and development as expected, since a particular reference condition (i.e. before treatment) was used for comparison. At least fifty DEGs associated with plant defence were identified in both resistant genotypes in comparison to the susceptible genotype (**Supplementary Table S5**), post inoculation. These included those related to cell-wall enforcement, phytohormone biosynthesis, NB-LRR protein, disease resistance protein and pathogen-related proteins, in addition to those involved in flavonoid biosynthesis and transcription factors including WRKY, bHLH and MYB. Among those genes, eight DEGs, chosen for their known roles in plant defence, were selected for subsequent gene expression validation (**Supplementary Table S7**) i.e. *RUST10*, *PR-1*, *RLP-1*, *PAL*, *RGA3*, *GLUCA*, *WAKL2* and *WRKY76*.

### 3.2 Validation of gene expression

Eight DEGs relating to plant defence were selected for gene expression studies by qPCR. Four candidate reference genes i.e. *MSD, NAD5, UBI* and *ACT1* were assessed to select the most stable gene to be used as a reference gene using RefFinder software. Based on the comprehensive stability ranking, *UBI* and *ACT1* were the least stable genes with scores of 3 and 4, respectively. *NAD5* was the second most stable gene (1.414), whilst *MSD* scored 1.189 on the index and was therefore selected as the reference gene for this study (**Supplementary Figure S2**).

**Figure 3:**
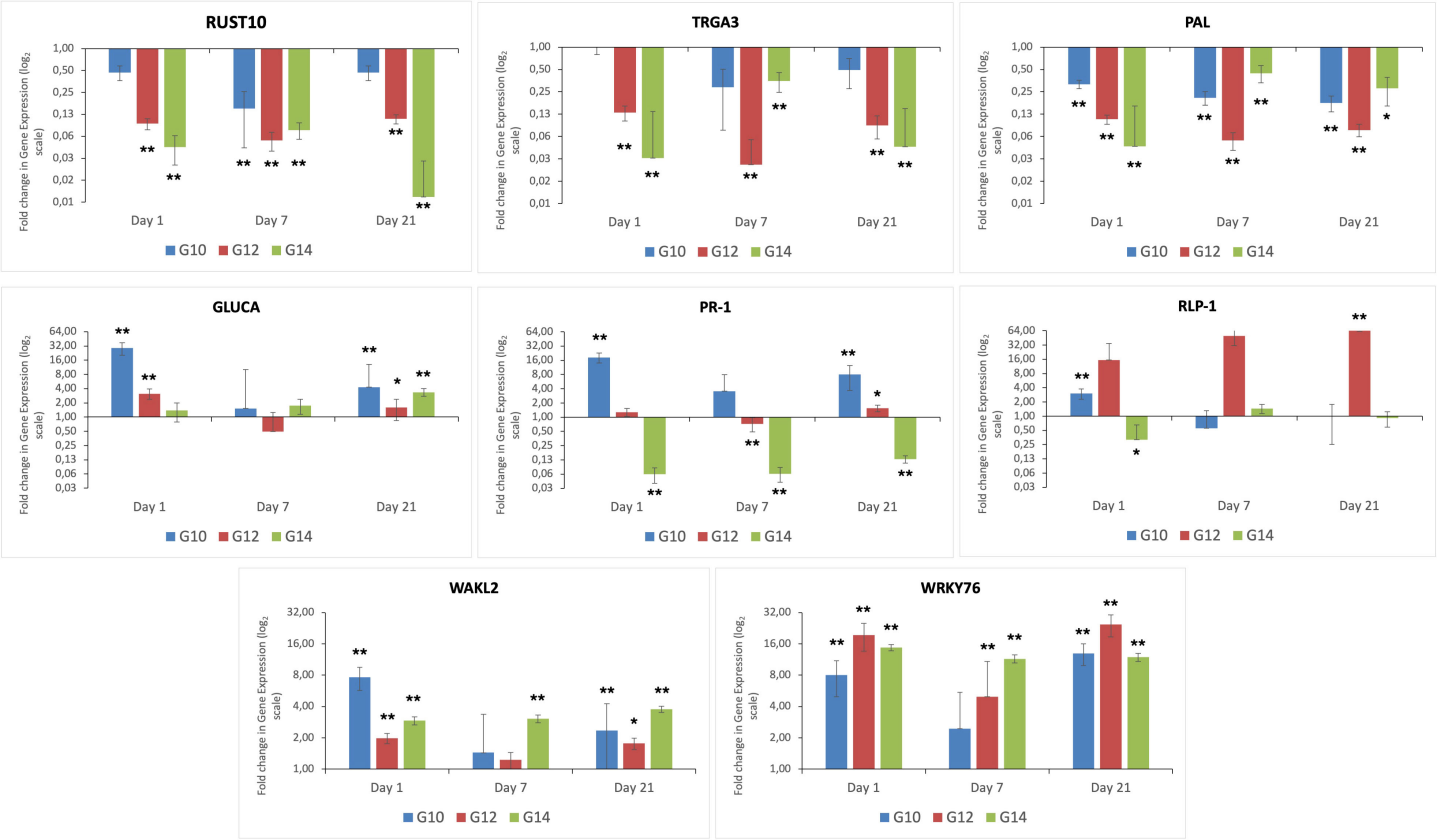
Differential expression of eight genes caused by infection with *C. oryzae* at different time points. Fold change is displayed relative to uninfected plants (day 0) of the same genotype. All ΔCt values were analysed using t-test, n = 9. **p < 0.01 and *p < 0.05 for difference in ΔCt value compared to reference condition.

Expression of the eight genes selected from the RNA-Seq DEG data was evaluated across the three different genotypes and time-points, both before exposure to the fungal pathogen (i.e. day 0) and post infection (days 1, 7 and 21), to identify differential responses over time between resistant and susceptible genotypes. These studies also enabled us to determine whether the response between the two resistant genotypes differed from one another (**Figure 3**). Genes encoding RUST10, RGA3 and PAL were down regulated at all time-points (i.e. day 1, 7 and 21) post inoculation in all genotypes, with the susceptible genotype G14 exhibiting the greatest levels of down-regulated expression, especially at day 1 post infection. In this susceptible genotype, genes encoding GLUCA, PR-1 and RLP-1 were either less expressed or down-regulated during infection, in comparison to either of the resistant genotypes. However, in keeping with both of the resistant genotypes (G10, G12), gene expression of WAKL2 and the transcription factor WRKY76 were increased in the susceptible genotype (G14) in response to inoculation. This finding would suggest that although these genes are known to be involved in plant defence, they failed to trigger an effective response against the fungal pathogen.

Overall, infection resulted in a down regulation of *RUST10* across all genotypes and timepoints, with the exception of G10 at day 1 and day 21 post infection. At day 7, expression of *RUST10* in G10 decreased to ~8-fold compared to the uninfected control plants at time zero. Expression of this gene in this genotype at day 7 post infection was significantly down-regulated in response to *C. oryzae* infection. Expression of *RUST10* in G12 was consistently down regulated at all time points investigated (3.2-fold), with expression being significantly different between infected and non-infected plants at all time points. The expression of *RUST10* in the susceptible genotype G14 was consistently down-regulated throughout the study, being decreased by 4.4 and 3.7-fold at day 1 and day 7-post infection, respectively. Expression of this gene was then further down regulated by up to 6.8-fold at 21 days post infection. Overall, the qRT-PCR validation data for all genes investigated in this study (**Figure 3**) were consistent with the RNA-seq data as shown in **Supplementary Table S5**.

## 4 Discussion

Two resistant genotypes (G10 and G12) and one susceptible genotype (G14) selected from previous nursery screening trials (Wibowo et al., 2023) were used to identify genes expressed during the asymptomatic (1 and 7 dai) and symptomatic (21 dai) phases of *C. oryzae* infection in oil palm seedlings under controlled conditions. RNA samples were sequenced using the Illumina NovaSeq 6000 platform to obtain high quality RNA sequencing data for downstream analysis. The experimental design permitted the identification of temporally differentially expressed genes within a genotype by comparing RNA isolated at 1, 7 and 21 dai with RNA isolated prior to application of *C. oryzae* spores. This design also enabled the differences in gene expression between the two resistant genotypes (G10 and G12) and the susceptible G14 genotype to be made.

The majority of transcriptomics studies investigating plant-pathogen interactions to date use samples collected over a relatively short period of time post treatment. For example, the response of oil palm to *Phytophthora palmivora*, responsible for causing oil palm bud rot, focussed on genes expressed during the asymptomatic phase, i.e. 120h post infection (Avila-Mendez et al., 2019);. In contrast, the current experimental design enabled the transcriptional response to be monitored both during the asymptomatic phase and during the later symptomatic phase for the three different genotypes. This allowed the identification of genes involved in the early and late response, including those encoding transcription factors, that play significant roles in the interaction of *C. oryzae*, with its host.

Prior to inoculation, the expression of genes in all genotypes was similar to one another, resulting in clustering, as demonstrated by principle component analysis (PCA) (**Figure 1B**). However, infected plants formed a distinct cluster after PCA, where the susceptible genotype clustered independently from the two resistant genotypes. These observations demonstrate that the induced response of the resistant genotypes at the transcriptional level differs to that of the susceptible genotype during pathogen infection.

In total 1,802 DEGs were identified when comparing all three genotypes at all time points between infected plants with non-infected healthy plants (day 0). However, only 172 DEGs were found when comparing between the resistant genotypes with the susceptible genotype, G14, as the control in the absence of infection. These results indicate that pathogen development from the asymptomatic (day 1 and day 7) to the symptomatic phase (day 21) caused major transcriptional reprogramming irrespective of the genotype, thus shedding light on the mechanisms employed by oil palm in defence against pathogen attack. These analyses identified many genes with known roles in plant defence, including those involved in phytohormone biosynthesis (SA, ABA, ethylene and brassinosteroid), cell-wall reinforcement (glycine-rich cell wall structural protein and wall-associated receptor kinase), and regulation of gene expression (transcription factors, WRKY, MYB, BIM, bHLH, and MYC). Furthermore, genes encoding peroxidases and chitinases, disease resistance protein (RGA1, RGA2, RGA3, and RGA4), leucine-rich repeat (LRR) proteins, pathogenesis-related (PR) proteins, glutathione S-transferases, cysteine-rich receptor-like kinases (CRKs) and thaumatin-like proteins (TLPs) were also shown to be involved in the defence response in the present study. These analyses were performed over a time course and, as such, several genes related to plant growth and development, were also identified as being differentially expressed.

Genes encoding RGA3 and RGA4, known to be related to disease resistance, were also differentially expressed between susceptible and resistant genotypes, as well as between time points. Plant resistance gene analogues (*RGAs*) play specific roles in the plant-pathogen interaction. *RGAs* are a class of potential resistance genes that have conserved domains and motifs and usually grouped as either nucleotide binding site leucine rich repeats (NBS-LRR) or transmembrane leucine rich repeats (TM-LRR) with two sub-classes i.e., receptor like kinases (RLK) and receptor like proteins (RLP). These canonical motifs allow simple and rapid identification from sequenced genomes (Sekhwal et al., 2015). A study in rice showed that RGA5/RGA4 form a functional heterodimer to recognize and trigger resistance to *Magnaporte oryzae* effectors through direct binding (Cesari et al., 2014). The disease resistance protein RGA3 has also been used to predict basal stem rot disease resistance in oil palm, based on the levels of transcripts and was filed as a patent (Tan et al., 2017). *De novo* assembly of 13 transcriptomic sequences in *Mangifera indica* has identified and characterized several RGAs including *RGA1, RGA2, RGA3* and *RGA4*, which can be utilized for disease resistance breeding (Lantican et al., 2020). In the current study, the *RGA3* gene in the susceptible genotype G14 was significantly more down-regulated at day 1 post treatment and at the symptomatic phase (day 21), than in the two resistant genotypes when compared with healthy plants before treatment. Plant disease resistance is also determined by the timely detection of the pathogen and by the rapid deployment of efficient plant defence reactions (Gullner et al., 2018). Generally late and weak host defence reactions result in host susceptibility and the current study indicates that the susceptible genotype has failed to maintain its defence against this fungal pathogen. However, study of *RGA3* in Ganoderma disease in oil palm showed higher gene expression levels in infected seedlings compared to healthy seedlings (Tan et al., 2017). These data suggest that *RGA3* and other *RGA* genes identified in this study can be validated and used in future breeding programmes to identify best candidate genes and levels of transcripts to select for varieties with enhanced resistance to leaf spot disease.

Similarly to *RGA3*, genes encoding leaf rust 10 disease-resistance locus receptor-like protein kinase (RUST10/Lrk10) and phenylalanine ammonia-lyase (PAL) at day 1 post treatment, showed greater down-regulation in the susceptible genotype than resistant genotypes. Schachermayr et al. (1997) showed that *Lrk10* co-segregates with gene *Lr10* and molecular markers derived from the *Lrk10* gene were also highly specific for the *Lr10* gene, which can be used for wheat breeding programmes to select against leaf rust (Schachermayr et al., 1997). A report involving hexaploid wheat, another monocotyledonous crop, revealed that the *Lr10* gene enhanced resistance to leaf rust when overexpressed by transgenesis. This gene shows homology both to the gene encoding for disease resistance protein RPM1 in *A. thaliana*, and also to *RGA* genes in rice and barley (Feuillet et al., 2003).

Phenylalanine ammonia-lyase (PAL) is an important factor for SA biosynthesis and therefore plant defence. A study in pepper (*Capsicum annuum*) during infection by *Xanthomonas campestris pv. vesicatoria* (Xcv) revealed that silencing of the *CaPAL1* gene increased plant susceptibility to Xcv infection and also significantly reduced SA accumulation, reactive oxygen species (ROS), and hypersensitive cell death (Kim and Hwang, 2014). Gene expression analysis in oil palm during Ganoderma infection demonstrated up-regulation of *PAL* genes in the infected seedlings compared to controls (Govender et al., 2017). *PAL* genes may act as an effective regulator of SA-dependent defence signalling against pathogens via enzymatic activity in the phenylpropanoid pathway (Kim and Hwang, 2014).

Differences in gene expression levels were observed for *PR-1, RLP-1* and *GLUCA* genes both at different time points and genotypes. The susceptible genotype, G14, demonstrated down-regulated gene expression in *PR-1* and *RLP-1* genes compared to up-regulated expression in the resistant genotypes G10 and G12. PR-1 protein is known for its fungicidal properties and is expressed by plants to fight pathogen invasion. A recent study in oil palm showed that a significant up-regulation of the *PR-1* gene occurred along with a reduction of *Ganoderma boninense* colonization (Bahari et al., 2018). The authors also proposed that the high expression of *PR-1* caused the removal of ergosterol, a primary metabolite and cell-wall component in *G. Boninense*, thus weakening the pathogen. RLPs and RLKs play important roles in plant immunity, growth, and development. Plants express a large number of RLKs and RLPs as pattern recognition receptors (PRRs), which detect pathogens as the first level of inducible defence (Tang et al., 2017). The resistant genotype G10, exhibited significantly higher levels of expression in these three DEGs, especially *GLUCA* and *PR-1*. Glucan 1,3-beta-glucosidase (GLUCA) is known to be involved in the metabolism of beta-glucan, the main structural component of the cell wall, and thus plays a vital role in cell wall stability. Based on our findings, we hypothesise that higher expression of *GLUCA* and *PR-1* genes at day 1 post treatment increases plant resistance to leaf spot disease, especially in the resistant genotype G10. WRKY transcription factors are known to play vital roles in the regulation of plant stress tolerance including pest, disease and environmental changes (Xiao et al., 2017; Alshegaihi, 2019). WRKY transcription factors have been shown to be differentially expressed in response to both reduced nitrogen and *Zymoseptoria tritici* invasion in wheat (Poll et al., 2020). In the present study *WAKL2* and *WRKY76* expression levels were highly up-regulated in both resistant and susceptible genotypes. Although these genes are involved in plant defence, their up-regulation in both susceptible as well as resistant genotypes suggests their involvement in the recognition of the pathogen rather than in defence *per se*, as the susceptible genotype G14 failed to trigger an effective response against leaf spot disease invasion.

The Cysteine-rich receptor-like kinases (CRKs) identified in the current study are likely to play a vital role in innate immunity as previously identified in Arabidopsis, where overexpression induced stomatal closure suggesting a strengthening of plant immunity (Yeh et al., 2015). CRKs are an important class of RLK that play key roles in disease resistance and cell death in plants (Romero et al., 2014). Studies on plant biotic stress have also demonstrated that specific glutathione S-transferases (*GSTs*) genes are up-regulated in response to microbial infections including bacterial, fungal and viral infections (Gullner et al., 2018). Several *GST* genes such as *GSTF1, U17, DHAR1* and *F12* were also identified in the current study; these genes are worthy of further investigation to better understand the mechanisms of leaf spot disease resistance in oil palm.

Interestingly, in this study, we found that the two resistant genotypes (G10 and G12), despite having different genetic backgrounds, exhibited similar gene expression patterns and disease resistance mechanisms. This finding suggests that the molecular basis of resistance to leaf spot disease is conserved in the oil palm.

## 5 Conclusions

RNA-seq studies have successfully identified a suite of genes related to oil palm defence against leaf spot disease. Eight genes, known to be involved in plant defence, were validated via gene expression studies with concordance to the RNA-seq results obtained. This knowledge will help inform oil palm breeding programmes to select for tolerance to leaf spot disease, as early and strong expression of these particular genes will increase the chance of suppressing pathogen invasion. However, it is important to continue to identify further genes involved in the pathogen response to select the best candidate genes for durable tolerance towards this specific pathogen. The best candidate genes, where there are distinct differences in the levels of expression between resistant and susceptible genotypes can be used for marker-assisted breeding. The current study lays the foundations for further studies to provide potential molecular targets for crop improvement.

## Data Availability

The datasets presented in this study can be found in online repositories. The names of the repository/repositories and accession number(s) can be found below: https://www.ncbi.nlm.nih.gov/, PRJNA914615.
