# Supplementary material for: Molecular basis of resistance to leaf spot disease in oil palm
Source: Front Plant Sci. 2024 Dec 9;15:1458346. doi: 10.3389/fpls.2024.1458346 (PMC11663676; doi:10.3389/fpls.2024.1458346)
Supplement: Supplementary file 2 [file Table1.docx]

Supplementary Material


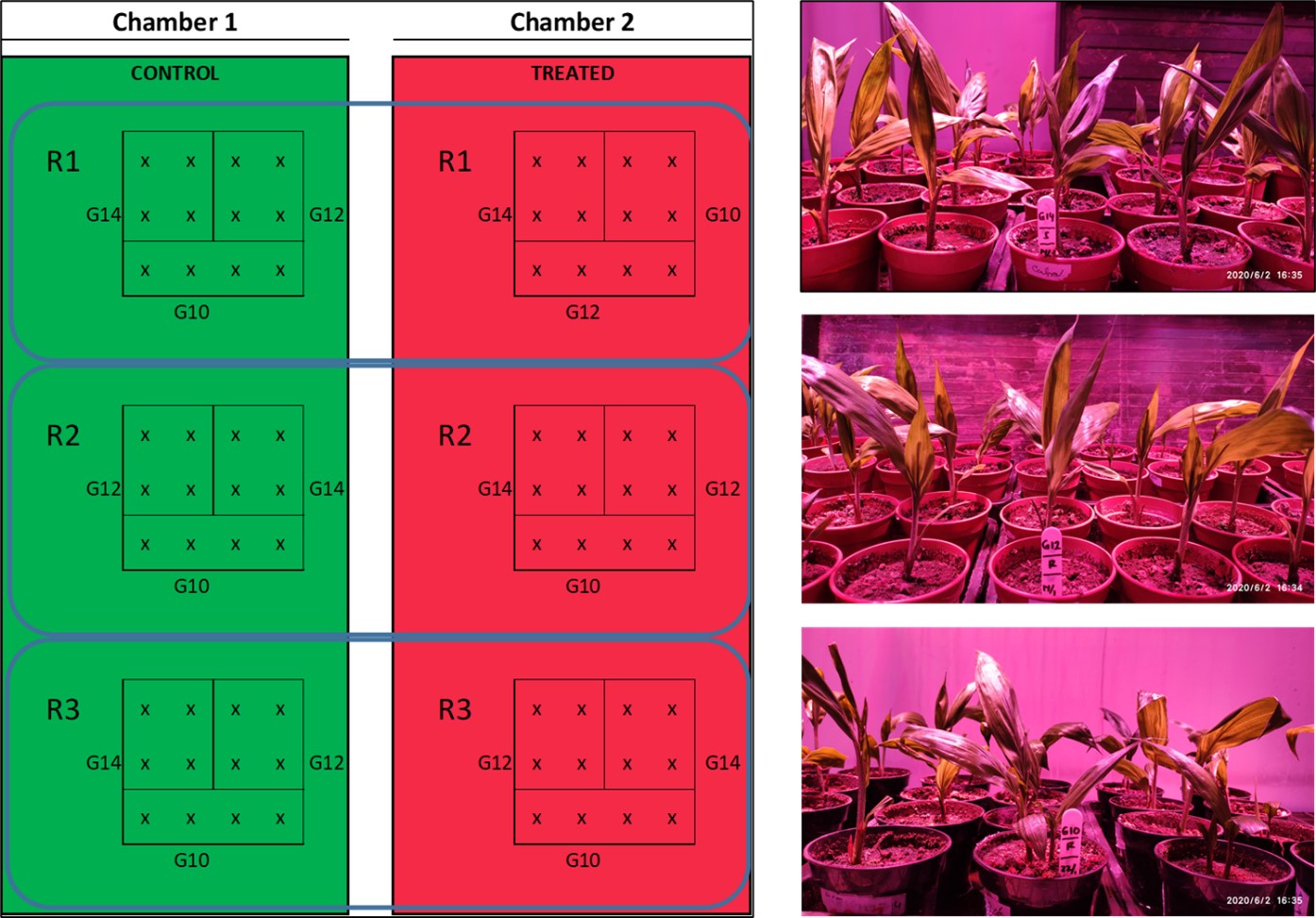


**Supplementary Figure S1.** Growth cabinets layout of experiment and the size of seedlings used for the experiment (5 months-old). G14 genotype (top right), G12 genotype (middle right), G10 genotype (bottom right). R1-R3 showed the replicate number.
