# Supplementary material for: Molecular basis of resistance to leaf spot disease in oil palm
Source: Front Plant Sci. 2024 Dec 9;15:1458346. doi: 10.3389/fpls.2024.1458346 (PMC11663676; doi:10.3389/fpls.2024.1458346)
Supplement: Supplementary file 3 [file Table2.docx]

Supplementary Material


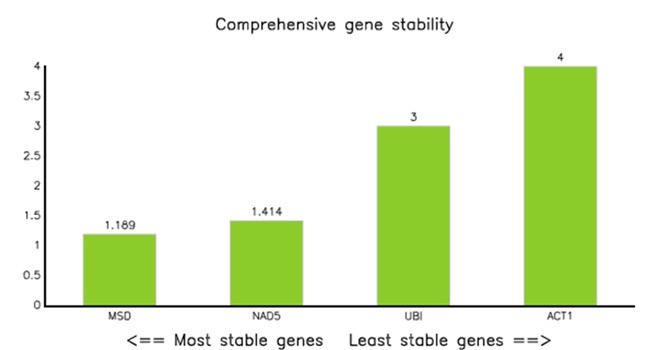


Supplementary Figure S2. Final ranking of four candidate reference genes assessed with RefFinder web-based software. The genes are MSD, NAD5, UBI and ACT1. MSD gene (on the left) was determined as the most stable and was selected as the reference gene.
