# Supplementary material for: Molecular basis of resistance to leaf spot disease in oil palm
Source: Front Plant Sci. 2024 Dec 9;15:1458346. doi: 10.3389/fpls.2024.1458346 (PMC11663676; doi:10.3389/fpls.2024.1458346)
Supplement: Supplementary file 4 [file Table3.docx]

Supplementary Material


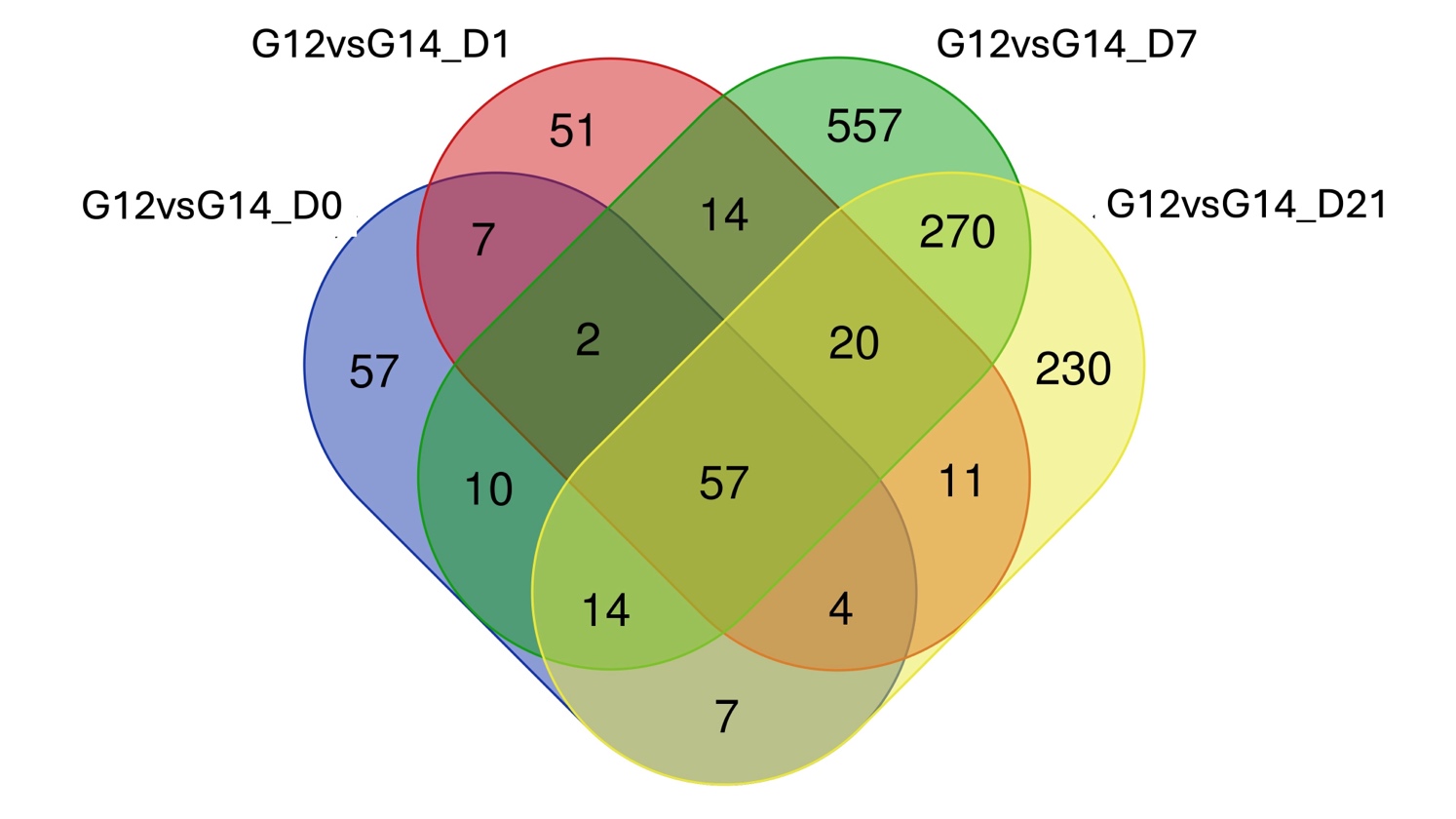


Supplementary Figure S3. The venn diagram of DEGs between the resistant genotype G12 and the susceptible genotype G14 at different time points.
