# Supplementary material for: Molecular basis of resistance to leaf spot disease in oil palm
Source: Front Plant Sci. 2024 Dec 9;15:1458346. doi: 10.3389/fpls.2024.1458346 (PMC11663676; doi:10.3389/fpls.2024.1458346)
Supplement: Supplementary file 5 [file Table4.docx]

Supplementary Material

Supplementary Table S1. List of DxP genotypes used in this experiment. The origins showed the genetic background of oil palm in both female and male sources.

| No | Genotype | Phenotype | Female Origin | Male Origin | Treatment |
| --- | --- | --- | --- | --- | --- |
| 1 | G10 | tolerant | Deli Dura Dabou 1 | La Me x SP540 3 | Control |
| 2 | G10 | tolerant | Deli Dura Dabou 1 | La Me x SP540 3 | Innoculated |
| 3 | G12 | tolerant | Deli Dura Dabou 2 | La Me x SP540 4 | Control |
| 4 | G12 | tolerant | Deli Dura Dabou 2 | La Me x SP540 4 | Innoculated |
| 5 | G14 | susceptible | Deli Dura 13 | SP540 5 | Control |
| 6 | G14 | susceptible | Deli Dura 13 | SP540 5 | Innoculated |
