# Supplementary material for: Molecular basis of resistance to leaf spot disease in oil palm
Source: Front Plant Sci. 2024 Dec 9;15:1458346. doi: 10.3389/fpls.2024.1458346 (PMC11663676; doi:10.3389/fpls.2024.1458346)
Supplement: Supplementary file 6 [file Table5.docx]

Supplementary Material

**Supplementary Table S2.** Primers sequences to amplify sections of housekeeping and targeted genes.

| **Target Gene** | **Forward Primers Sequence (5’-3’)** | **Reverse Primers Sequence (5’-3’)** |
| --- | --- | --- |
| NAD5 | GCT CCC CTT TAT TTG AAT ACC C | AAT AGT TAG AGA TGC CGC AAG C |
| MSD | AGA GCG CCA TCA AGT TCA AT | CTT ATC CAG AGC AAG CCA CAC |
| ACT1 | GTT GTC GCT CCA CCC G | GCA GGA CCA CAT TCA TCA TA |
| UBI | CAC TTT GGA GGT CGA GTC GT | GTG GAG GGT GGA CTC CTT T |
| RUST10 | CCG AAG TCT TCT CCA GGA ATT T | CTG ACG TCC TCC AAC CAT TT |
| TRGA3 | ATG CTG CCA CGA CTA GAT TAC | GAA GTA GGT GGA CAA GGA AGA G |
| GLUCA | CGA CAC CCA CTA CTA CAA TCT C | CTT GGA TCG CTG GTT GTT AAT G |
| PR1 | TTG CTC ACC ATT CTC CAT CTC | GGA TTG TTT CCT AGG CTT CAC TA |
| RLP1 | TGT AGA ACT CGT CAG CAA AGG | AGC GGG ATG TTT CCA GTT AG |
| PAL | GTA CTG ACC ATC GGA GCT AAT G | TCT GCA TCA AAG GGT AGG TAA AG |
| WRKY76 | AGA CTT ACG AGA GGA CCG ATA G | GTG ATC TTG GGC TTC GTA TCT T |
| WAKL2 | CCC AGT GGA TGC CAA GAT ATA A | TGT AGC TCC CAA GGG AAT TAA G |
