# Supplementary material for: Molecular basis of resistance to leaf spot disease in oil palm
Source: Front Plant Sci. 2024 Dec 9;15:1458346. doi: 10.3389/fpls.2024.1458346 (PMC11663676; doi:10.3389/fpls.2024.1458346)
Supplement: Supplementary file 7 [file Table6.docx]

Supplementary Material

**Supplementary Table S3.** List of RNA samples for RNA sequencing.

| No. | Name | Treatment | Concentration (ng/µl) | Volume (µl) | Total amount (µg) | Integrity value | Sample QC Results |
| --- | --- | --- | --- | --- | --- | --- | --- |
| 1 | 1001 | G10·Day 0 | 108 | 48 | 5.18 | 7.3 | Pass |
| 2 | 1002 | G10·Day 0 | 151 | 42 | 6.34 | 7 | Pass |
| 3 | 1003 | G10·Day 0 | 99 | 40 | 3.96 | 6.9 | Pass |
| 4 | 1201 | G12·Day 0 | 177 | 40 | 7.08 | 7 | Pass |
| 5 | 1202 | G12·Day 0 | 156 | 41 | 6.40 | 7.1 | Pass |
| 6 | 1203 | G12·Day 0 | 153 | 41 | 6.27 | 6.9 | Pass |
| 7 | 1401 | G14·Day 0 | 208 | 39 | 8.11 | 7 | Pass |
| 8 | 1402 | G14·Day 0 | 101 | 40 | 4.04 | 6.8 | Pass |
| 9 | 1403 | G14·Day 0 | 178 | 39 | 6.94 | 7.5 | Pass |
| 10 | 1011 | G10·Day 1 | 128 | 39 | 4.99 | 7 | Pass |
| 11 | 1012 | G10·Day 1 | 76 | 39 | 2.96 | 6.9 | Pass |
| 12 | 1013 | G10·Day 1 | 140 | 37 | 5.18 | 7 | Pass |
| 13 | 1211 | G12·Day 1 | 113 | 39 | 4.41 | 7.1 | Pass |
| 14 | 1212 | G12·Day 1 | 55 | 40 | 2.20 | 7.5 | Pass |
| 15 | 1213 | G12·Day 1 | 73 | 39 | 2.85 | 7 | Pass |
| 16 | 1411 | G14·Day 1 | 46 | 40 | 1.84 | 7.4 | Pass |
| 17 | 1412 | G14·Day 1 | 116 | 39 | 4.52 | 7.3 | Pass |
| 18 | 1413 | G14·Day 1 | 53 | 39 | 2.07 | 6.7 | Pass |
| 19 | 1071 | G10·Day 7 | 129 | 40 | 5.16 | 6.8 | Pass |
| 20 | 1072 | G10·Day 7 | 137 | 40 | 5.48 | 6.6 | Pass |
| 21 | 1073 | G10·Day 7 | 222 | 40 | 8.88 | 7.2 | Pass |
| 22 | 1271 | G12·Day 7 | 120 | 38 | 4.56 | 6.8 | Pass |
| 23 | 1272 | G12·Day 7 | 73 | 39 | 2.85 | 7.3 | Pass |
| 24 | 1273 | G12·Day 7 | 82 | 38 | 3.12 | 6.9 | Pass |
| 25 | 1471 | G14·Day 7 | 110 | 38 | 4. 18 | 6.9 | Pass |
| 26 | 1472 | G14·Day 7 | 111 | 38 | 4.22 | 7.8 | Pass |
| 27 | 1473 | G14·Day 7 | 84 | 39 | 3.28 | 7.1 | Pass |
| 28 | 1031 | G10·Day 21 | 74 | 39 | 2.89 | 7 | Pass |
| 29 | 1032 | G10·Day 21 | 98 | 40 | 3.92 | 6.8 | Pass |
| 30 | 1033 | G10·Day 21 | 95 | 40 | 3.80 | 6.9 | Pass |
| 31 | 1231 | G12·Day 21 | 109 | 42 | 4.58 | 7 | Pass |
| 32 | 1232 | G12·Day 21 | 82 | 39 | 3.20 | 7 | Pass |
| 33 | 1233 | G12·Day 21 | 60 | 39 | 2.34 | 7.1 | Pass |
| 34 | 1431 | G14-Day 21 | 119 | 36 | 4.28 | 7.1 | Pass |
| 35 | 1432 | G14-Day 21 | 93 | 40 | 3.72 | 7.3 | Pass |
| 36 | 1433 | G14-Day 21 | 113 | 38 | 4.29 | 6.9 | Pass |
