# Supplementary material for: Molecular basis of resistance to leaf spot disease in oil palm
Source: Front Plant Sci. 2024 Dec 9;15:1458346. doi: 10.3389/fpls.2024.1458346 (PMC11663676; doi:10.3389/fpls.2024.1458346)
Supplement: Supplementary file 8 [file Table7.docx]

Supplementary Material

**Supplementary Table S4.** RNA-seq Data Quality Summary.

| No. | Treatment | Sample | Raw reads  (bp) | Raw data (Gbp) | Read mapping (%) | Error(%) | Q20(%) ^a^ | Q30(%) ^b^ | GC (%) |
| --- | --- | --- | --- | --- | --- | --- | --- | --- | --- |
| 1 | G10·Day 0 | R1001 | 43579296 | 6.5 | 94.71 | 0.03 | 97.41 | 92.81 | 49.50 |
| 2 | G10·Day 0 | R1002 | 43879242 | 6.6 | 94.63 | 0.03 | 97.33 | 92.63 | 49.59 |
| 3 | G10·Day 0 | R1003 | 44996948 | 6.7 | 94.47 | 0.03 | 97.29 | 92.57 | 49.33 |
| 4 | G12·Day 0 | R1011 | 43400840 | 6.5 | 95.82 | 0.03 | 97.31 | 92.56 | 48.77 |
| 5 | G12·Day 0 | R1012 | 44277998 | 6.6 | 96.39 | 0.03 | 97.34 | 92.64 | 48.30 |
| 6 | G12·Day 0 | R1013 | 43881456 | 6.6 | 95.88 | 0.03 | 97.34 | 92.64 | 48.53 |
| 7 | G14·Day 0 | R1031 | 39428606 | 5.9 | 94.23 | 0.03 | 97. 17 | 92. 37 | 49.08 |
| 8 | G14·Day 0 | R1032 | 39236590 | 5.9 | 95.23 | 0.03 | 97. 16 | 92. 18 | 48.89 |
| 9 | G14·Day 0 | R1033 | 45685560 | 6.9 | 95.88 | 0.03 | 97.06 | 92.09 | 48.60 |
| 10 | G10·Day 1 | R1071 | 465542 14 | 7.0 | 95.57 | 0.03 | 97.41 | 92.87 | 49.30 |
| 11 | G10·Day 1 | R1072 | 44375440 | 6.7 | 95.26 | 0.03 | 97.24 | 92.49 | 49.36 |
| 12 | G10·Day 1 | R1073 | 44386030 | 6.7 | 95.36 | 0.03 | 97.42 | 92.87 | 49.69 |
| 13 | G12·Day 1 | R1201 | 49910962 | 7.5 | 92.70 | 0.03 | 97.54 | 93.24 | 49.59 |
| 14 | G12·Day 1 | R1202 | 44008350 | 6.6 | 95.26 | 0.03 | 97.34 | 92.72 | 49.41 |
| 15 | G12·Day 1 | R1203 | 44692932 | 6.7 | 94.78 | 0.03 | 97.47 | 92.92 | 49.24 |
| 16 | G14·Day 1 | R1211 | 44292662 | 6.6 | 95.85 | 0.03 | 97.42 | 92.78 | 48.59 |
| 17 | G14·Day 1 | R1212 | 44271250 | 6.6 | 95.90 | 0.03 | 97.09 | 92. 18 | 48.77 |
| 18 | G14·Day 1 | R1213 | 43868486 | 6.6 | 95.26 | 0.03 | 97.42 | 92.83 | 48.95 |
| 19 | G10·Day 7 | R1231 | 43703324 | 6.6 | 95.76 | 0.03 | 97.25 | 92.51 | 48.85 |
| 20 | G10·Day 7 | R1232 | 43136074 | 6.5 | 94.28 | 0.03 | 97.22 | 92.49 | 48.57 |
| 21 | G10·Day 7 | R1233 | 42936816 | 6.4 | 95.45 | 0.03 | 97.00 | 92.02 | 48.70 |
| 22 | G12·Day 7 | R1271 | 44861032 | 6.7 | 95.30 | 0.03 | 97.31 | 92.61 | 49.33 |
| 23 | G12·Day 7 | R1272 | 44441004 | 6.7 | 95.40 | 0.03 | 97. 11 | 92. 11 | 49.32 |
| 24 | G12·Day 7 | R1273 | 43798802 | 6.6 | 94.85 | 0.03 | 97.35 | 92.69 | 48.57 |
| 25 | G14·Day 7 | R1401 | 44439832 | 6.7 | 95.23 | 0.03 | 97. 36 | 92.74 | 49.22 |
| 26 | G14·Day 7 | R1402 | 43869026 | 6.6 | 95.81 | 0.03 | 97.03 | 91.94 | 49.94 |
| 27 | G14·Day 7 | R1403 | 47340104 | 7.1 | 95.65 | 0.03 | 97. 36 | 92.69 | 49.00 |
| 28 | G10·Day 21 | R1411 | 44558822 | 6.7 | 95.26 | 0.03 | 97.47 | 92.90 | 49. 15 |
| 29 | G10·Day 21 | R1412 | 459 19928 | 6.9 | 94.85 | 0.03 | 97.21 | 92.44 | 48.92 |
| 30 | G10·Day 21 | R1413 | 41934842 | 6.3 | 94.63 | 0.03 | 97.51 | 92.98 | 49.50 |
| 31 | G12·Day 21 | R1431 | 45013782 | 6.8 | 94.81 | 0.03 | 97.28 | 92.60 | 50.18 |
| 32 | G12·Day 21 | R1432 | 45814104 | 6.9 | 95.22 | 0.03 | 97. 11 | 92. 19 | 48.97 |
| 33 | G12·Day 21 | R1433 | 45197352 | 6.8 | 94.42 | 0.03 | 97. 35 | 92.71 | 49.03 |
| 34 | G14-Day 21 | R1471 | 44504760 | 6.7 | 94.95 | 0.03 | 97. 53 | 93.06 | 49. 19 |
| 35 | G14-Day 21 | R1472 | 45344982 | 6.8 | 94.70 | 0.03 | 97. 14 | 92.33 | 49.54 |
| 36 | G14-Day 21 | R1473 | 43192116 | 6.5 | 95.46 | 0.03 | 97.20 | 92.40 | 48.47 |

^a^ A quality score of 20 (Q20) represents an error rate of 1 in 100

^b^ A quality score of 30 (Q30) represents an error rate of 1 in 1000
