# Supplementary material for: Molecular basis of resistance to leaf spot disease in oil palm
Source: Front Plant Sci. 2024 Dec 9;15:1458346. doi: 10.3389/fpls.2024.1458346 (PMC11663676; doi:10.3389/fpls.2024.1458346)
Supplement: Supplementary file 9 [file Table8.docx]

Supplementary Material

**Supplementary Table S5.** List of 50 DEGs related to plant defence identified in both resistant genotypes at three different time points post inoculation

| **Gene Code** | **Product** |
| --- | --- |
| Eg001 | thaumatin-like protein 1 |
| Eg002 | glycine-rich cellwall structural protein 2 |
| Eg003 | wall-associated receptor kinase 4 |
| Eg004 | flavonol 3-sulfotransferase |
| Eg005 | MLO-like protein 1 |
| Eg006 | peroxidase 3 |
| Eg007 | probable leucine-rich repeat receptor-l ke protein kinase At1g35710 |
| Eg008 | transcription factor MYC2 |
| Eg009 | receptor-like protein EIX2 |
| Eg010 | pathogenesis-related protein 1 |
| Eg011 | peroxidase 12-like |
| Eg012 | putative glycine-rich cellwall structural protein 1 |
| Eg013 | probable cysteine desulfurase |
| Eg014 | chitinase-like protein 1 |
| Eg015 | polygalacturonase QRT3 |
| Eg016 | transcription factor MYB61 |
| Eg017 | chalcone synthase |
| Eg018 | pathogen-related protein |
| Eg019 | LRR receptor-like serine/ threonine-protein kinase ERECTA |
| Eg020 | transcription factor MYB20 |
| Eg021 | ethylene-responsive transcription factor ERF038 |
| Eg022 | peroxidase 4 |
| Eg023 | probable polygalacturonase At1g80170 |
| Eg024 | putative disease resistance protein At3g14460 |
| Eg025 | probable disease resistance protein At4g27220 |
| Eg026 | transcription factor bHLH75 |
| Eg027 | dihydroflavonol 4-reductase |
| Eg028 | LRR receptor-l ke serine/ threonine-protein kinase FLS2 |
| Eg029 | transcription factor BIM1 |
| Eg030 | brassinosteroid LRR receptor kinase BRL1-like |
| Eg031 | cysteine-rich receptor-l ke protein kinase 6,transcript variant X1 |
| Eg032 | probable LRR receptor-l ke serine/ threonine-protein kinase At3g47570 |
| Eg033 | thaumatin-like protein 1b |
| Eg034 | phenylalanine ammonia-lyase |
| Eg035 | probable WRKY transcription factor 72 |
| Eg036 | wall-associated receptor kinase 2-like |
| Eg037 | pathogenesis-related protein 5 |
| Eg038 | transcription factor bHLH18 |
| Eg039 | protein PYRICULARIA ORYZAE RESISTANCE 21 |
| Eg040 | flavonol sulfotransferase-like |
| Eg041 | peroxidase 72 |
| Eg042 | transcription repressor OFP3-like |
| Eg043 | pathogenesis-related protein 1 |
| Eg044 | LRR receptor-like serine/ threonine-protein kinase ERL1 |
| Eg045 | putative disease resistance protein RGA3 |
| Eg046 | transcription factor MYB36 |
| Eg047 | pathogenesis-related protein PRB1-3 |
| Eg048 | ethylene-responsive transcription factor ERF003 |
| Eg049 | disease resistance protein RGA2-l ke |
| Eg050 | Polygalacturonase inhibitor |
