# Supplementary material for: Molecular basis of resistance to leaf spot disease in oil palm
Source: Front Plant Sci. 2024 Dec 9;15:1458346. doi: 10.3389/fpls.2024.1458346 (PMC11663676; doi:10.3389/fpls.2024.1458346)
Supplement: Supplementary file 10 [file Table9.docx]

Supplementary Material

**Supplementary Table S7.** List of selected DEGs related to plant defence, cell-wall hardening, environmental stress and phytohormone synthesis. The first eight genes were used for gene expression study. Log2 fold change > 2.0 and adjusted p-value > 0.05 were used as threshold.

| **Gene Code** | **Gene ID** | **logFC** | **adj.P.Val** | **Product** |
| --- | --- | --- | --- | --- |
| RUST10 | 105041568 | -4.6874 | 4.41E-41 | leaf rust 10 disease-resistance locus receptor-like protein kinase |
| TRGA3 | 105039989 | -2.2539 | 9.03E-05 | putative disease resistance protein RGA3 |
| GLUCA | 114912642 | 2 .5898 | 2.15E-16 | probable glucan 1,3-beta-glucosidase A |
| PR-1 | 105057434 | 3.8536 | 3.44E-08 | pathogenesis-related protein 1 |
| RLP-1 | 114913098 | -3.502 | 3.18E-16 | receptor-like protein 1 |
| PAL | 105055673 | -2.6659 | 2.75E-12 | phenylalanine ammonia-lyase |
| WAKL2 | 105054852 | 4.3256 | 3.54E-28 | wall-associated receptor kinase 2-like |
| WRKY76 | 105060191 | 3.8745 | 1.33E-14 | WRKY transcription factor WRKY76 |
| DRP460 | 105046867 | 5.0564 | 5.59E-49 | putative disease resistance protein At3g14460 |
| SIRK | 105041856 | 4.1086 | 5.32E-81 | senesence·induced receptor-I ike serine/threonine-protein kinase |
| WRKY70 | 105044827 | 3.2244 | 4.00E-07 | probable WRKY transcription factor 70 |
| GRGA3 | 105061556 | -3.0724 | 3.28E-18 | putative disease resistance protein RGA3 |
| RLK7 | 105061016 | 2 .3619 | 2.49E-10 | receptor-like protein kinase 7 |
| PR4 | 105042178 | 6.1006 | 2.451SE-22 | pathogenesis-related protein PR-4 |
| CRK6 | 105052281 | 4.121 | 2.78E-34 | cysteine-rich receptor-like protein kinase 6 |
| ERF1B | 105059101 | 3.8698 | 1.91E-07 | ethylene·responsive transcription factor 18 |
| TGLUC | 105045344 | 5.3635 | 2 .2615E-14 | glucan endo-1,3-beta-glucosidase |
| GGLUC | 105033963 | -2.1946 | 2.92E-05 | glucan endo-1,3-beta-glucosidase-like |
| GAPDH | 105051363 | 4.0723 | 5.54E-48 | glyceraldehyde·3-phosphate dehydroge nase 2 |
| ABA | 105058241 | -3.2002 | 9.93E-20 | abscisic acid 8'-hydroxylase 3 |
|  |  |  |  |  |
